# γδ T Lymphocytes in the Diagnosis of Human T Cell Receptor Immunodeficiencies

**DOI:** 10.3389/fimmu.2015.00020

**Published:** 2015-01-29

**Authors:** Beatriz Garcillán, Ana V. M. Marin, Anaïs Jiménez-Reinoso, Alejandro C. Briones, Miguel Muñoz-Ruiz, María J. García-León, Juana Gil, Luis M. Allende, Eduardo Martínez-Naves, María L. Toribio, José R. Regueiro

**Affiliations:** ^1^Department of Immunology, Complutense University School of Medicine and Hospital 12 de Octubre Health Research Institute, Madrid, Spain; ^2^Centro de Biología Molecular Severo Ochoa, Consejo Superior de Investigaciones Científicas and Universidad Autónoma, Madrid, Spain; ^3^Division of Immunology, Hospital General Universitario, Gregorio Marañón and Health Research Institute, Madrid, Spain; ^4^Division of Immunology, Hospital Universitario 12 de Octubre and Health Research Institute, Madrid, Spain

**Keywords:** TCR, immunodeficiency, diagnosis, γδ, T lymphocyte

## Introduction

Human T cell receptor (TCR) immunodeficiencies (TCRID) are rare autosomal recessive disorders caused by mutations affecting TCR, CD3, or CD247 chains, which share developmental, functional, and TCR expression defects (1). Their rapid diagnosis is fundamental for patient survival and early hematopoietic stem cell transplantation. Here, we propose that studying γδ T cells, which are often neglected, can be helpful for a timely diagnosis. We thus offer a diagnostic flowchart and some lab tricks based on published cases.

## γδ T Cell and TCR Physiopathology

γδ T lymphocytes are a minor subset (1–10%) of human peripheral blood T cells. Most (>70%) are CD4^−^CD8^−^ [double negative (DN)], some (30%) are CD8^+^CD4^−^ and very few (<1%) are CD4^+^CD8^−^ [CD8^+^ or CD4^+^ single positive (SP), respectively]. Most γδ T cells in adults express Vδ2/Vγ9 TCR variable regions (65–90%), the rest being mostly Vδ1^+^, some Vδ3^+^ or Vδ5^+^, all with different Vγ chains (2). As peripheral blood γδ T cells are scarce, their over-representation is more conspicuous than their under-representation, which is very rarely reported and normally associated to a single subset, such as Vδ2^+^ in granulomatosis (3) or aging (4). Indeed, no selective γδ T cell immunodeficiency (ID) has been reported to date, although absence of γδ T cells has been described together with other lymphocyte derangements in rare primary ID (5). The clinical significance of increased γδ T cells, defined as >10% of peripheral blood T lymphocytes (6), requires clarification in several diseases including infection, autoimmunity, cancer, and primary ID.

**Figure 1:**
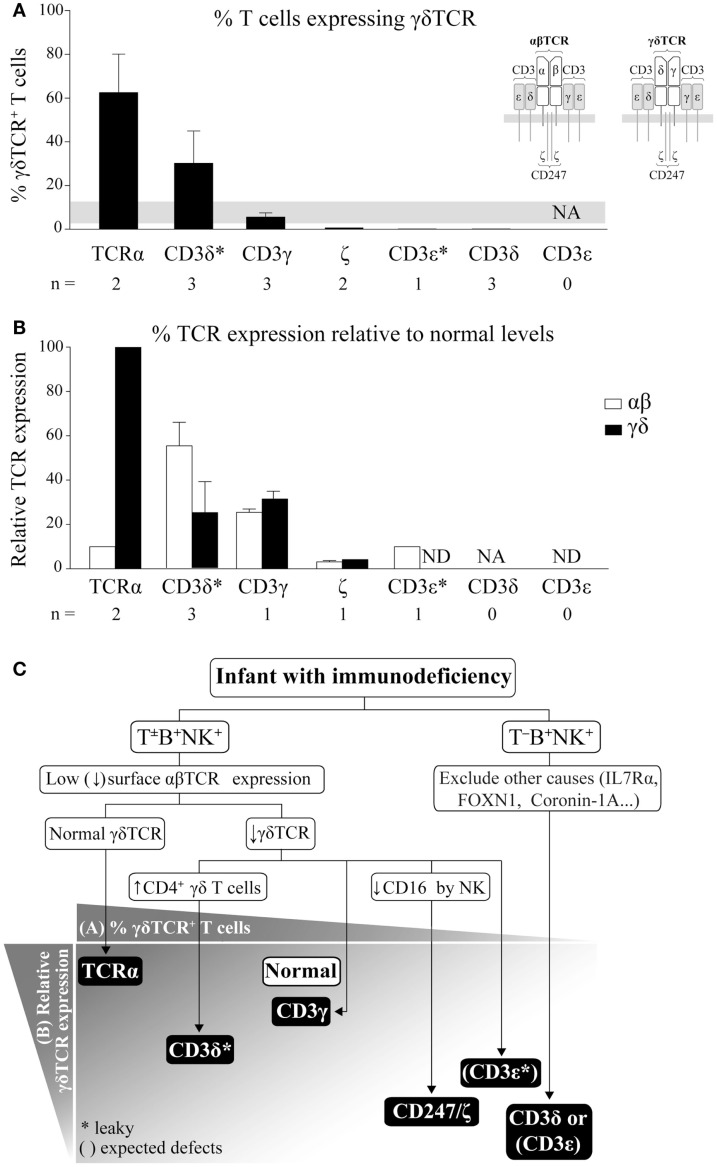
**γδ T cells in TCRID**. **(A)** Proportion of γδ T cells within the T cell compartment. The percentage of γδ T cells (mean ± SEM) was defined as γδTCR^+^ using 11F2, IMMU510, or anti-TCRδ-1 monoclonal antibody (mAb) within the T lymphocyte gate (defined as CD3^+^) and ordered from left to right in decreasing values. The gray band indicates the normal range for infants (8). Inset: human TCR isotypes. NA: not analyzable (no T cells); *: leaky mutations (partial defects); *n*: number of patients for which data was available. **(B)** % TCR surface expression (mean ± SEM) in γδ or αβ T cells relative to healthy donors. TCR surface expression was determined by flow cytometry using different anti-CD3 mAb, γδ T cells were identified as in **(A)** and αβ T cells as γδTCR^−^CD3^+^ or CD4^+^ cells. ND: not determined. **(C)** Our suggested TCRID diagnostic flowchart using absolute lymphocyte counts for T^−^B^+^NK^+^ or T^±^B^+^NK^+^ phenotype and basic flow cytometry data (top) to point to the most likely culprit TCR chain (bottom). TCR chains are represented by black boxes arranged according to the proportion of γδ T cells from **(A)** and their surface TCR expression relative to normal controls from **(B)**. The white box indicates normal value. *: as in **(A)**. Brackets represent expected defects, as γδ T cells values were not available in these TCRID.

The human γδTCR (Figure 1A inset) is an octameric protein complex composed of three heterodimers (TCRγ/TCRδ, CD3γ/CD3ε, and CD3δ/CD3ε) and a single CD247 homodimer (also termed ζ/ζ). The complex can be abbreviated as γδTCR/γεδεζζ. The TCRγ/TCRδ heterodimer contains variable regions, which allow for antigen recognition, while the other three dimers are invariant and are required for surface TCR expression and for intracellular propagation of the recognition signal (7). Therefore, defects in any chain would expectedly impact γδTCR expression and γδ T cell selection and function.

## γδ T Cells in TCRID

αβ T cells have been extensively studied in TCRID. In contrast, γδ T cells have been frequently ignored, in part due to their scarcity but also to the lack of markers other than the TCR to identify them when TCR expression is reduced, as is the case in TCRID. Although their functions are still debated, we believe that their accurate study (relative numbers, Figure 1A, TCR expression, Figure 1B, and main subsets) can help to diagnose TCRID, as reviewed below and summarized in a practical diagnostic flowchart in Figure 1C.

TCRα deficient patients showed combined ID and autoimmune features due to a selective block in αβ T cell development, as TCRα is part of the TCRα/TCRβ (αβTCR, Figure 1A inset) antigen-binding heterodimer (9). In contrast, the γδTCR was unaffected, as demonstrated by normal surface expression (Figure 1B), which allowed for normal absolute but increased relative numbers of γδ T cells (Figure 1A). This is unique among TCRID and thus a useful feature in the diagnostic flowchart (Figure 1C). Such γδ T cells were proposed to be in part protective from infections in the two reported patients. Indeed, γδ T cells are involved in immune responses against a variety of pathogens including virus, bacteria, and parasites, whereas still other act as antigen-presenting cells (10) or B cell helper cells (11). Their beneficial effects *in vivo* have found recent unexpected recognition in haploidentical allogeneic hematopoietic cell transplantation after depletion of αβ T and B cells (12), which showed that γδ T cells did not cause graft vs. host disease and may have helped with host immune maintenance and recovery. The fact is that, compared to other complete TCRID, symptoms in both TCRα deficient patients appeared rather late (6 and 15 months of age) and transplantation took place very late (6–7 years of age).

Similar to TCRα deficient patients, patients with partial CD3δ deficiency (CD3δ* in Figure 1) due to a leaky splicing mutation showed strongly reduced αβ T cell numbers and normal absolute but high relative numbers of γδ T cells (Figure 1A), although with low surface TCR expression [(13) and Figure 1B]. In contrast to TCRα deficiency, partial CD3δ deficiency showed early severe combined ID (SCID) features and required very early transplantation (before 2 years of age), thus their γδ T cells were not protective, perhaps as a consequence of their impaired TCR expression and function (13). Unexpectedly, partial CD3δ deficiency caused a stronger impact in γδ (Figure 1B) than in αβTCR surface expression (25 vs. 55% relative to controls (13). A detailed study of their γδ T cells showed an enrichment in a subset of otherwise rare CD4^+^ γδ T cells, which exhibited an activated phenotype and were refractory to further TCR stimulation (14). This CD4-expressing γδ T cell subset seems to be pathognomonic for partial CD3δ deficiency, since: (i) it has been ascertained in three of three tested patients with this condition and (ii) it was not found in other TCRID (14, 15). Its developmental origin deserves further comment. αβ and γδ T cells differentiate within the thymus from a late DN common progenitor (16). In humans, development of most γδ T cells seems to mimic that of αβ T cells: from DN progenitors through a CD4^+^CD8^+^ double positive (DP) pathway (17, 18), to DN and either CD4^+^ or CD8^+^ SP populations. DN and CD8^+^ SP γδ T cells are minor intrathymic subsets but become the major γδ T cell subsets in the periphery, while CD4^+^ SP are the main intrathymic subset, followed by DP (19). Notably, the last two subsets can be found in peripheral blood in pathological conditions, and most bone marrow and peripheral blood γδ T cells from patients with γδ T cell acute lymphoblastic leukemia are either CD4^+^ SP or DP (20). Thus, we believe that the 10-fold enrichment of CD4^+^ SP γδ T cells observed in patients with partial CD3δ deficiency is due to low TCR-dependence for positive selection of CD4^−^ γδ T cells and disrupted negative selection of CD4^+^ γδ T cells (14).

CD3γ deficient patients, most of which showed mild ID (21), had normal numbers of polyclonal peripheral blood γδ T cells [absolute and relative, (22) and Figure 1A] with low surface TCR [around 30% of control levels (22) and Figure 1B], similarly to their αβ T cell counterparts (23), likely with an abnormal γδTCR/δεδεζζ stoichiometry. Despite their high homology, the invariant CD3γ and δ chains show different roles in human vs. mouse γδ T cell development. Indeed, CD3γ-deficient mice exhibited a severe γδ T cell developmental block (24).

γδ T cells were studied in only two of three reported CD247 deficient patients (21). The patients showed SCID features and reduced absolute and relative γδ T cells numbers (Figure 1A). Surface γδTCR expression was also reduced (4% vs. healthy controls, Figure 1B). The number of αβ T cells was only slightly reduced despite their similarly reduced surface TCR, with all reported cases showing reduced numbers of CD4^+^ T cells but normal or high numbers of CD8^+^ T cells (21, 25–27).

A single patient with partial CD3ε deficiency (28–30) showed very low surface αβTCR expression (10% of normal levels, Figure 1B, CD3ε*), mild ID, normal CD8^+^, and reduced CD4^+^ (αβ) T cells, but no γδ T cells as determined with the anti-TCRδ-1 monoclonal antibody (mAb) (Figure 1A). We have however considered for Figure 1C that surface γδTCR expression might have been similar to αβTCR expression.

Three studied CD3δ deficient patients [out of 16 reported, all with severe T cell lymphopenia and SCID (31, 32)], showed a few circulating CD3^+^ T cells, which were DN but γδTCR^−^(33). γδTCR^+^ cells were indeed undetectable by flow cytometry in peripheral blood or by immunohistochemistry in the thymus, lymph nodes, spleen, or gut. However, gene microarray analysis and protein expression of patient thymocytes showed increased levels of TCRγ and TCRδ transcripts and proteins (33), which could be interpreted as presence and thus significant selection of γδ T cells unable however to leave the thymus, perhaps due to insufficient surface TCR compared to partial CD3δ deficiency.

Finally, γδ T cells have not been studied in SCID patients with complete CD3ε deficiency (31). Nevertheless, given their severe T cell lymphopenia, we can safely presume for Figure 1C that they were absent.

In summary, the proportion of γδ T cells within total T lymphocytes (Figure 1A) and the level of surface γδ vs. αβTCR expression (Figure 1B), as well as the severity of lymphopenia (T^−^B^+^NK^+^ or T^±^B^+^NK^+^ phenotype), can be used to generate a practical TCRID diagnostic flowchart (Figure 1C). For instance, if an infant has SCID and no T cells but normal B and NK cell numbers (T^−^B^+^NK^+^ phenotype) and other causes have been ruled out, CD3δ or CD3ε deficiency should be considered (Figure 1C). In contrast, if some T cells are present (T^±^B^+^NK^+^ phenotype) and γδ TCR expression is low, TCRα deficiency can be ruled out. If CD16 expression by NK cells is normal, CD247 deficiency can be excluded, and the presence or absence of high absolute numbers of CD4^+^ γδ T cells will rule out CD3γ or partial CD3δ deficiency, respectively.

## Lab Tricks to Identify αβ and γδ T Cells in TCRID

When surface TCR expression is low, αβ T cells can be identified by the expression of CD4 or CD8αβ (i.e., CD8^bright^) within the lymphoid subset (23), whereas γδ T cells are identified only by expression of the γδTCR. We have reported that most CD3^+^ cells within normal DN lymphocytes are γδ T cells (34), and this may also help in certain TCRID.

Despite their reduced numbers and surface TCR expression, an appropriate multicolor flow cytometry approach can help to identify γδ T cells in TCRID. To avoid underestimation due to low TCR surface expression, we recommend: (i) the use of bright fluorochromes such as PE, PE-Cy5.5, PE-Cy7, or APC, rather than FITC, (ii) an appropriate choice of CD3 mAb such as UCHT-1, F101.01, or S4.1 due to their high signal-to-noise ratio in TCRID, (iii) two-color stainings with CD3 and γδTCR mAb, which can also help to single out γδ T cells as a DP subset, and (iv) to avoid mixing αβTCR and CD3 mAb, as they sometimes compete (UCHT-1, for instance).

CD4 and CD8 expression by γδ T cells should also be tested to rule out partial CD3δ deficiency (see above). CD4, CD8, γδTCR (IMMU510 or 11F2), and CD3 (UCHT-1 or S4.1) is a useful combination, to this end. Lastly, intracellular stainings for invariant TCR chains has been shown to be useful to identify T cells expressing very low surface TCR, such as those with CD247 (21) or partial CD3δ deficiency (14).

## Conclusion and Perspectives

Human γδ T lymphocytes are still puzzling in terms of development, function, and TCR stoichiometry in ways that mouse models do not wholly recapitulate. Human TCRID share defects in T cell development and function and in TCR expression. While their αβ T cells have been studied in detail, γδ T cells have been frequently ignored, in part due to their scarcity and to the lack of appropriate markers to identify them when TCR expression is reduced. Here, pooling published studies, we proposed some technical tricks to identify γδ T cells in TCRID patients and made the point that their careful analysis can help to inform a rapid differential diagnosis using a flowchart, with clinical benefit.

## Conflict of Interest Statement

The authors declare that the research was conducted in the absence of any commercial or financial relationships that could be construed as a potential conflict of interest.

## Abbreviations

DN, double negative; DP, double positive; ID, immunodeficiency; mAb, monoclonal antibody; SCID, severe combined immunodeficiency; SP, single positive; TCR, T cell receptor; TCRID, T cell receptor immunodeficiencies.
